# Identification of distinct metabolic characteristics of pneumonia in type 2 diabetes mellitus

**DOI:** 10.1002/ctm2.303

**Published:** 2021-02-04

**Authors:** Jingwen Huang, Ying Xie, Daoyi Yuan, Lingxi Guo, Jieming Qu, Min Zhou

**Affiliations:** ^1^ Department of Respiratory and Critical Care Medicine, Ruijin Hospital Shanghai Jiao Tong University School of Medicine Shanghai China; ^2^ Institute of Respiratory Diseases Shanghai Jiao Tong University School of Medicine Shanghai China; ^3^ Department of Pharmacology and Chemical Biology Shanghai Jiao Tong University School of Medicine Shanghai China

Dear Editor,

Pneumonia is the leading infectious cause of death worldwide, with approximately 3 million annual casualties based on the World Health Organization data.^1^ The incidence and short‐ or long‐term mortality of lower respiratory tract infections in patients with type 2 diabetes mellitus (T2DM) are much higher than those of non‐diabetic patients.^2^, ^3^, ^4^ To our knowledge, the number of patients in China with T2DM was 88.5 million in the year 2017. The elderly have the highest prevalence of T2DM in China and are also more susceptible to pneumonia.^5^, ^6^ Early detection and intervention in pneumonia patients with T2DM are crucial; however, few specific targets for this purpose have been identified.

In this study, we conducted ultraperformance liquid chromatography–tandem quadrupole time‐of‐flight mass spectrometry (UPLC‐QTOF/MS) metabolome profiling of serum samples and a transcriptomic validation of peripheral blood mononuclear cells (PBMCs) to determine the metabolic characteristics of pneumonia in T2DM patients (Figure 1A, study scheme). All methods are detailed in Materials and Methods section of the Supporting Information.

**FIGURE 1:**
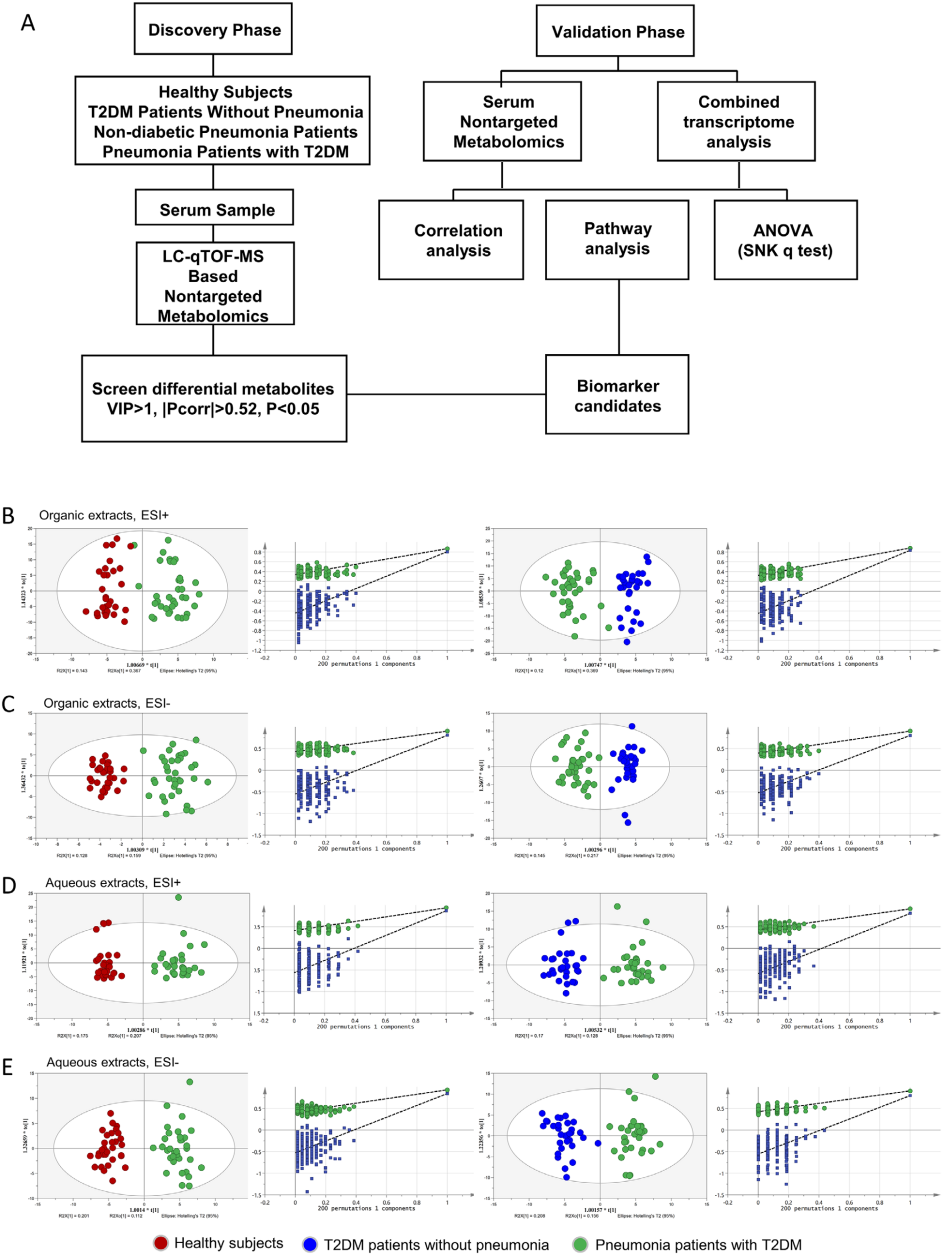
The Scheme of exploration on metabolic profile of pneumonia patients with T2DM. (A) Scheme of this study. (B‐E) The OPLS‐DA score in four modules of metabolomics analysis via UPLC‐QTOF/MS: organic extracts in the (B) ESI+ mode and (C) ESI− mode; aqueous extracts in the (D) ESI+ mode and (E) ESI− model. Permutation testing was carried out for the validation of the OPLS‐DA model. Circles in red represent samples from healthy control, circles in blue represent samples from T2DM patients without pneumonia, and circles in green represent samples from pneumonia patients with T2DM.

Thirty‐six pneumonia patients with T2DM, 31 non‐diabetic pneumonia patients, 31 T2DM patients without pneumonia, and 31 healthy controls without pneumonia or T2DM were enrolled between March 2018 and December 2018 in the discovery set. Twenty‐five pneumonia patients with T2DM, 31 non‐diabetic pneumonia patients, 27 T2DM patients without pneumonia, and 27 healthy controls were enrolled between January 2019 and December 2019 in the validation set. The ages and sexes of the subjects in each group of the discovery and validation sets were balanced as closely as possible. Except for serum glucose level, there were no significant differences in the parameters between pneumonia patients with or without T2DM (Table 1). However, pneumonia patients with T2DM required longer hospital stays to achieve clinical stability compared with non‐diabetic pneumonia patients in both the discovery set (Median [interquartile range,IQR] length of stay, 19.5 [13.3–26.0] days vs 12.0 [8.0–20.0] days; p = 0.009) and the validation set (Median [IQR] length of stay, 25.0 [14.0–36.0] vs 19.0 [10.0–27.0]; p = 0.108). This was consistent with previous findings in hospitalized patients with diabetes.^7^

**TABLE 1:** Clinical information of the subjects enrolled in the metabolomics study

Baseline characteristic	Discovery set				Validation set			
	Healthy subjects (n = 31)	T2DM patients (n = 31)	Non‐diabetic pneumonia patients (n = 31)	Pneumonia patients with T2DM (n = 36)	Healthy subjects (N = 27)	T2DM patients (n = 27)	Non‐diabetic pneumonia patients (n = 31)	Pneumonia patients with T2DM (n = 25)
Age, years	72.0 (65.0–79.0)	73.0 (71.0–79.0)	68.0 (62.0–82.0)	69.0 (65.0–77.0)	63.0 (60.0–68.0)	63.0 (62.0–65.0)	67.0 (54.0–74.0)	65.0 (56.5–76.5)
Male (n,%)	21 (68)	27 (87)	25 (81)	25 (69)	19 (70)	19 (70)	16 (52)	21 (84)
BMI, kg·m^−2^	25.2 (23.5–25.9)	23.7 (22.2–25.3)	22.6 (20.8–26.3)	24.5 (22.6–26.8)	23.6 (20.7–27.0)	24.7 (21.4–27.1)	21.9 (21.0–23.7)	24.2 (22.1–26.1)
Leucocyte (× 10^9^/L)	6.8 (5.6–8.3)	5.9 (5.1–7.1)	8.1 (4.7–11.7)^a^	6.5 (4.9–10.3)^a^	5.8 (4.8–6.4)	5.7 (5.0–6.9)	7.3 (5.8–13.3)^a^	7.5 (5.6–10.1)^a^
Neutrophil (× 10^9^/L)	4.0 (3.2–4.9)	3.4 (2.9–4.1)	5.9 (2.9–8.3)^a^	5.4 (3.3–8.5) ^a^	3.4 (2.7–3.8)	3.2 (2.9–4.1)	5.1 (3.4–11.1)^a^	4.8 (3.4–7.4)^a^
Lymphocyte(× 10^9^/L)	2.0 (1.6–2.4)	1.5 (1.3–1.8)	1.1 (0.8–1.4)	1.0 (0.6–1.4)	1.7 (1.22–2.05)	1.9 (1.6–2.20)	1.2 (0.8–1.9) ^a^	1.4 (1.0–2.1)
NLR	2.0 (1.7, 2.5)	2.3 (1.8, 2.9)	4.9 (2.3, 10.1)^a^	5.9 (3.4, 10.4)^a^	1.9 (1.8, 2.6)	2.0 (1.5, 2.3)	5.0 (2.3, 12.3)^a^	2.7 (1.9, 5.8)
Neutrophil, %	60.0 (53.0–65.0)	61.4 (56.7–66.7)	75.9 (60.2–84.0)^a^	76.1 (69.3–84.4)^a^	60.0 (55.0–65.0)	60.0 (51.9–63.7)	73.8 (62.2–84.5)^a^	67.4 (59.0–78.3)^a^
Lymphocyte,%	29.0 (25.0–33.0)	26.8 (23.7–32.1)	16.1 (8.3–26.2)^a^	13.7 (8.3–22.3)^a^	31.0 (21.0–35.0)	31.8 (27.5–37.1)	18.5 (7.3–27.7)^a^	23.0 (8.9–30.0)^a^
Albumin (g/L)	45.0 (42.0–50.0)	46.0 (44.3–47.8)	32.0 (27.0–35.0)^a^	32.0 (26.3–34.6)^a^	46.0 (40.0–51.0)	44.0 (42.8–46.0)	35.0 (28.0–40.0)^a^	32.0 (30.0–37.5)^a^
Creatinine (μmol/L)	70.0 (64.0–79.0)	84.0 (74.0–88.0)	74.0 (59.0–85.0)	72.0 (62.0–96.5)	75.0 (68.0–80.0)	70.5 (60.0–78.8)	75.0 (66.0–85.0)	71.0 (60.5–81.0)
Urea nitrogen (mmol/L)	4.6 (3.2–6.4)	5.6 (5.1–7.1)	5.3 (4.0–7.3)^a^	5.4 (3.4–7.5)	4.9 (3.6–5.6)	5.2 (4.1–6.4)	5.8 (4.4–7.2)^a^	6.5 (4.7–7.5)^a^
Glucose (mmol/L)	4.9 (4.4–5.4)	7.5 (6.8–9. 1)^a^	5.5 (5.0–6.4)	9.4 (7.2–11.8)^a^, ^b^	5.1 (4.7–5.4)	8.8 (8.3–10.2)^a^	5.4 (4.9–6.6)	8.2 (6.1–12.5)^a^, ^b^
D‐Dimer (mg/L)			1.5 (0.3–4.1)	1.4 (0.6–3.2)			1.2 (0.3–3.6)	1.0 (0.3–3.6)
Fg (g/L)			4.6 (3.2–6.2)	4.1 (2.7–5.3)			3.8 (2.8–5.7)	3.9 (2.5–6.1)
CURB‐65 score (n, %) 0–1 2 ≥3			21(68) 9(29) 1(3)	22(61) 13(36) 1(3)			19(61) 11(36) 1(3)	17(68) 5(20) 3(12)
Length of stay			12.0 (8.0–20.0)	19.5 (13.3–26.0)^b^			19.0 (10.0–27.0)	25.0 (14.0–36.0)

Data are presented as median (interquartile range) for continuous variables and n (%) for categorical variables.

^a^ Represented p < 0.05 in the comparison of clinical parameters in T2DM without pneumonia, non‐diabetic pneumonia patients, pneumonia patients with T2DM versus healthy subjects without pneumonia or T2DM by one‐way ANOVA.

^b^ Represented p < 0.05 in the comparison of clinical parameters in non‐diabetic pneumonia patients versus pneumonia patients with T2DM.

Unsupervised principal component analysis (PCA) and orthogonal partial least‐squares discriminant analysis (OPLS‐DA) were performed to characterize the metabolite patterns corresponding to the maximum separation between the included subgroups. As illustrated by the PCA score plot, patients with pneumonia were clearly separated from those without pneumonia under four different modules (Figure 2A). The OPLS‐DA model revealed a significant differential metabolic profile between pneumonia patients with T2DM and healthy controls or T2DM patients without pneumonia (Table S1; Figures 1B‐1E). According to the criteria of a variable importance in the projection > 1.0 and absolute covariance p(corr) value > 0.52 in the OPLS‐DA model and p < 0.05 in the univariate analysis between pneumonia patients with T2DM and T2DM patients without pneumonia or healthy controls, 91 differential metabolites were ultimately identified (Figure S1, Table S2). Most were different types of lipids, including intermediate metabolites of glycerophospholipids and arachidonic acids (Figure 2B; Table S3).

**FIGURE 2:**
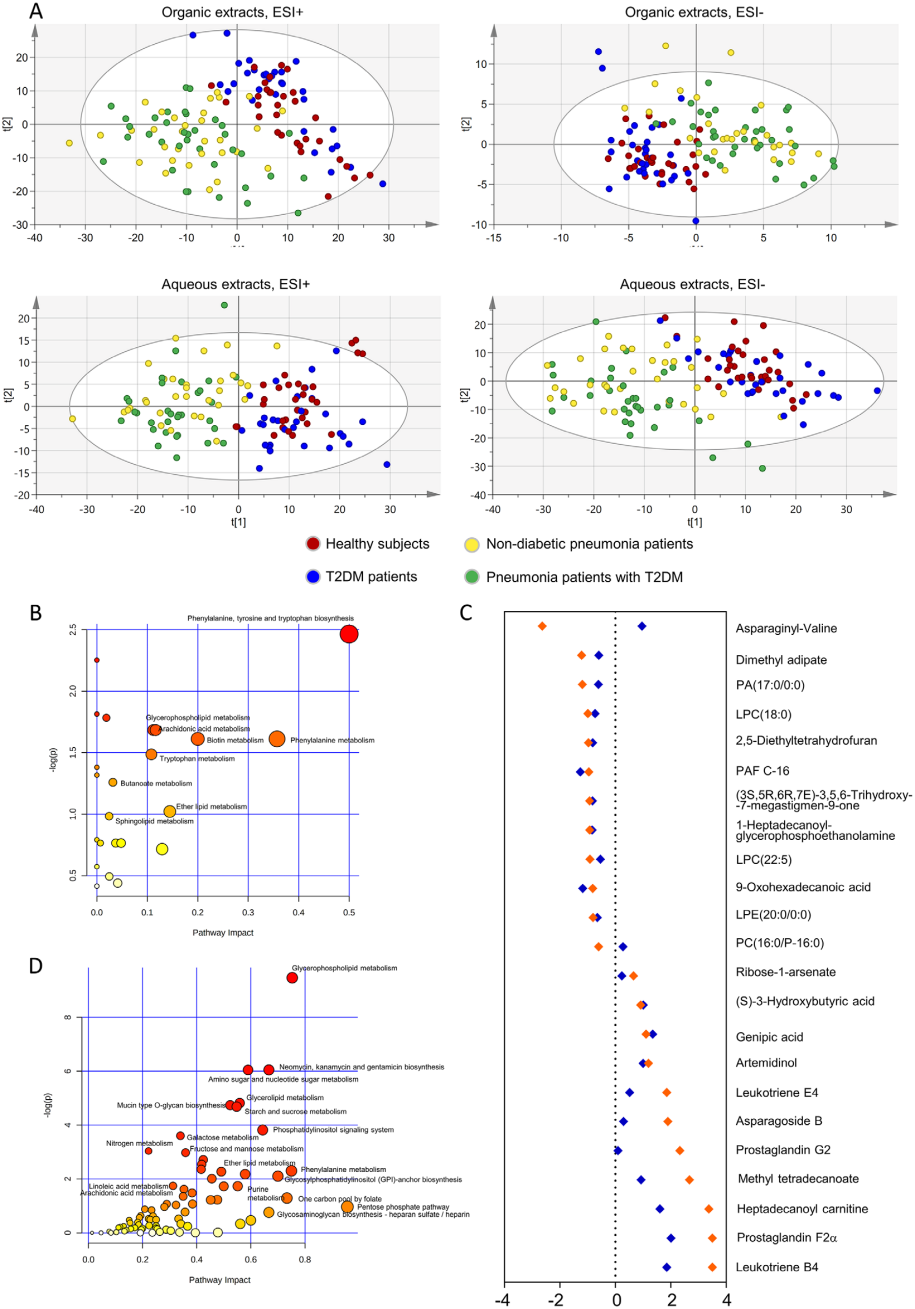
Pathway and enrichment analysis with the differential metabolites. (A) Score plot from PCA of organic extracts in the ESI+ mode and ESI− mode and of aqueous extracts in the ESI+ mode and ESI− model. Healthy subjects (red circle), T2DM without pneumonia (blue circle), non‐diabetic pneumonia patients (yellow circle) and pneumonia patients with T2DM (green circle). (B) Pathway analysis with MetaboAnalyst on the differential metabolites between pneumonia patients with T2DM and healthy subjects or T2DM patients. Each circle in is a representative of a biological pathway and the size of the circle is enumerated based on the importance. (C) Cleveland plots of the differential metabolites between pneumonia patients with T2DM versus healthy subjects whose AUC > 0.85, fold change > 1.5 and p‐value < 0.05. The whole set of metabolites was ranked according to fold changes in the discovery set. The results of the discovery set were shown in orange and the validation set were in blue. (D) The joint pathway analysis by MetaboAnalyst on differential metabolites and genes between pneumonia patients with T2DM group and healthy subject group. Each circle in is a representative of a biological pathway, and the size of the circle is enumerated based on the importance.

There were 23 metabolites in the discovery set that met the criteria, with an area under the curve > 0.85, and an absolute log2 fold change > 0.585, which we considered the most significant differential metabolites between pneumonia patients with T2DM and T2DM patients without pneumonia or healthy controls (Figure 2C, Tables S4 and S5). The heatmap showed that arachidonic acid intermediates were significantly upregulated in pneumonia patients with T2DM compared to healthy controls, while phospholipid metabolites were significantly downregulated (Figure 3A). Strong correlations were found within groups of related metabolites, especially arachidonic acids and phospholipids (Figure 3B).

**FIGURE 3:**
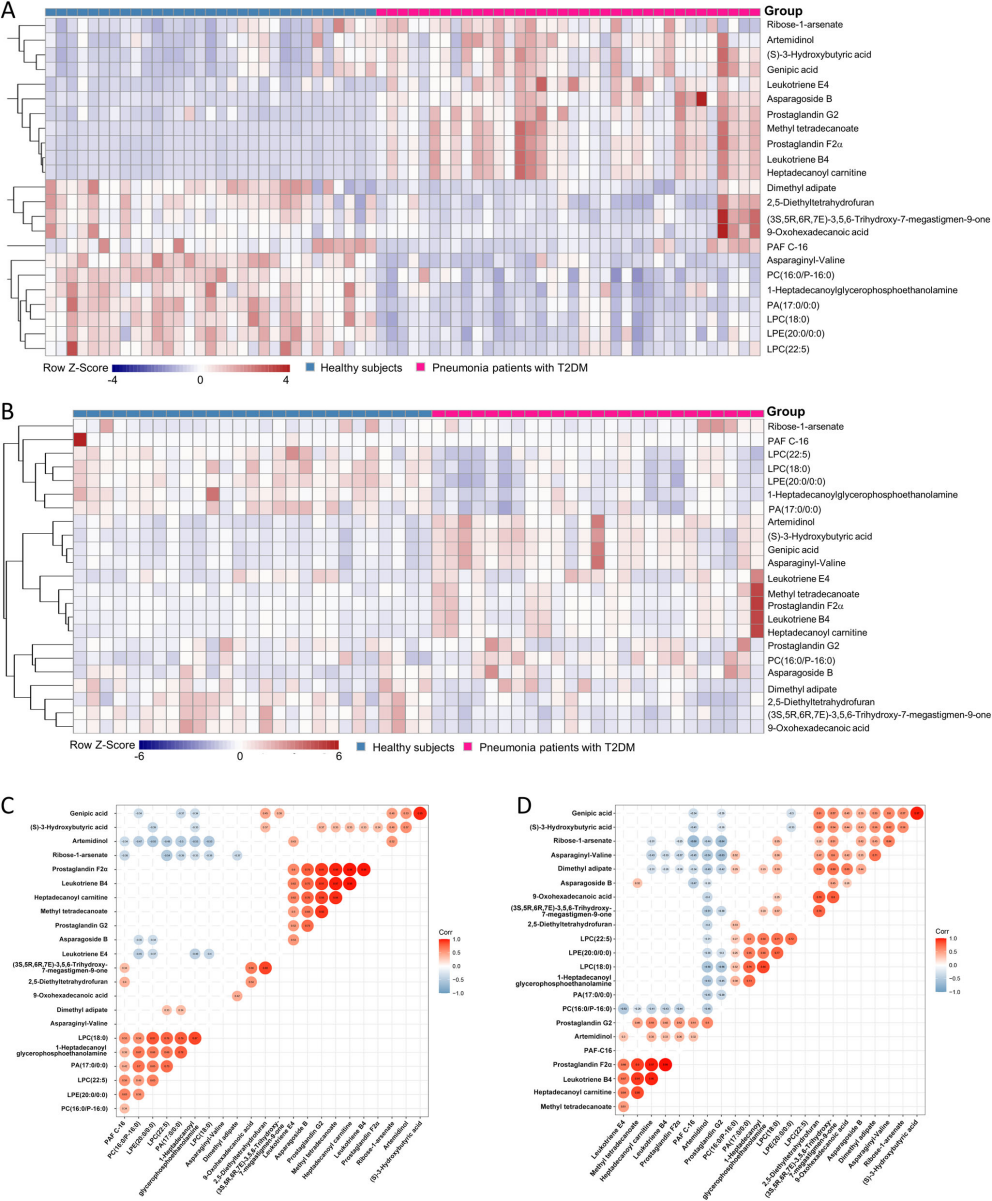
Distribution and correlation analysis of the identified significant differential metabolites between pneumonia patients with T2DM and healthy subjects. (A) The heatmap of the amount of the 23 novel metabolic candidates in pneumonia patients with T2DM group and healthy subject group in the discovery set. (B) The heatmap of the amount of the 23 novel metabolic candidates in pneumonia patients with T2DM group and healthy subject group in the validation set. Correlation coefficients between the 23 most significant differential metabolites in pneumonia patients with T2DM group (C) in the discovery set and (D) in both the discovery and validation set. Only these coefficients of p value < 0.05 were shown.

Transcriptomic analysis was conducted on PBMC samples to validate the results of the untargeted metabolomics (Table S6, patient characteristics). The results of the gene set enrichment analysis indicated that the most significantly altered pathways were those related to IL‐6 signalling, oxidative stress, and fatty acid metabolism, which were all upregulated in pneumonia patients with T2DM compared to healthy subjects and T2DM patients without pneumonia (Figure S2). The combined analysis of metabolomic and transcriptomic results suggested that glycerophospholipid metabolism was the most affected metabolic pathway by pneumonia in T2DM patients (Figure 2D, Table S7, Figure S3).

Based on the relative intensities of the metabolites from the normalised profiling data, one‐way ANOVA post hoc tests were applied to reveal the significant differences in the 91 differential metabolites between each group. The Student‐Newman‐Keuls q results showed that the serum lysophosphatidylcholine (LPC) (18:0) level was significantly decreased in pneumonia patients regardless of diabetes history, and was further decreased in pneumonia patients with T2DM compared to non‐diabetic pneumonia patients (Figure 4A). A significant difference in the LPC (18:0) value was still observed after adjustment of serum glucose levels (Table S8). The LPC (18:0) value was significantly lower in the higher SMART‐COP score group than in the 0–1 SMART‐COP score group in pneumonia patients with T2DM (Figure 4B). Meanwhile, serum LPC (18:0) levels were positively correlated with serum albumin levels (r = 0.378, p = 0.004) (Figure 4C), but negatively correlated with neutrophil to lymphocyte ratio (NLR) (r = ‐0.332; p = 0.012) in pneumonia patients with T2DM (Figure 4D). Studies have suggested that there is a positive association between the decrease in plasma LPC levels and lethality in septic patients.^8^ In vivo studies also proved that LPC, especially LPC (18:0), markedly increased bactericidal activity. The mechanism may be enhancement of the bactericidal response of innate immunity.^9^, ^10^ The small population size and lack of adjustment for multiple comparisons in the identification of differential metabolites are limitations of our study.

**FIGURE 4:**
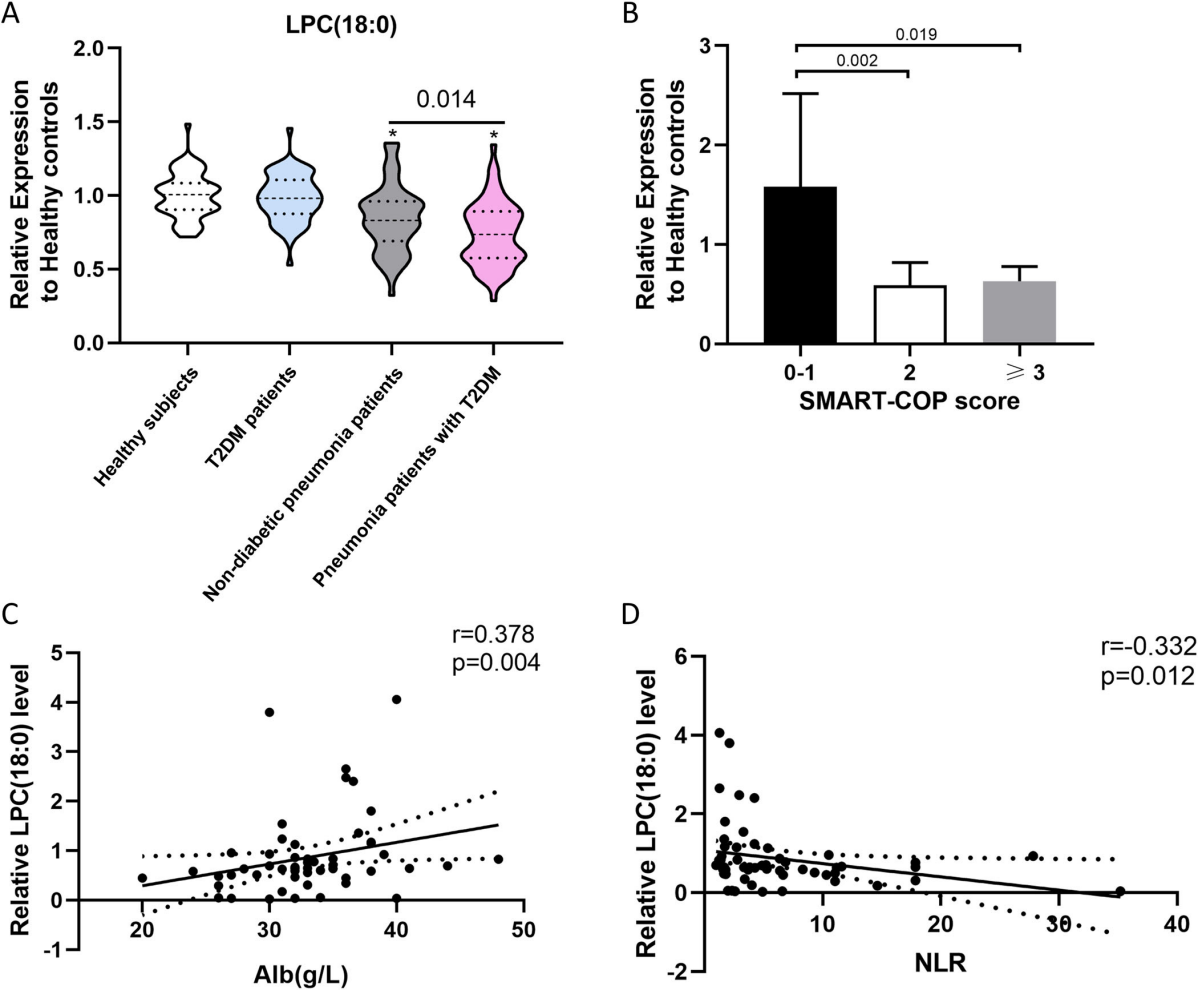
The serum LPC (18:0) level for discriminating non‐diabetic pneumonia patients and pneumonia patients with T2DM. (A) Decreasing trend of serum LPC (18:0) level in pneumonia patients with T2DM in relation to non‐diabetic pneumonia patients. (B) Relative serum LPC (18:0) level in pneumonia patients with T2DM based on SMART‐COP score. (C) Positive correlation between serum LPC (18:0) level and serum albumin level in pneumonia patients with T2DM. (D) Negative correlation between serum LPC (18:0) level and neutrophil‐to‐lymphocyte ratio in pneumonia patients with T2DM. ^*^p < 0.05 represents significant difference for the group compared with the healthy control group Abbreviation: NLR, neutrophil‐to‐lymphocyte ratio.

In conclusion, the results presented here demonstrate that the levels of phospholipid metabolites are decreased in patients with pneumonia. Serum LPC (18:0) levels were significantly lower in pneumonia patients with T2DM than in non‐diabetic pneumonia patients balanced by age, gender, and CURB‐65 score, although overall metabolic changes were similar between the two groups. The LPC (18:0) level was positively associated with the favorable prognosis indices among pneumonia patients with T2DM. Our findings provide new perspectives for further studies to validate LPC, especially LPC (18:0), as a useful biomarker for improving the poor outcome of pneumonia in T2DM patients.

## CONFLICT OF INTEREST

The authors declare that there is no conflict of interest that could be perceived as prejudicing the impartiality of the research reported.

## ETHICS APPROVAL AND CONSENT TO PARTICIPATE

The Ruijin Hospital Ethics Committee approved the study protocol and received verbal informed consent because patients involved in the study, the data, and serum sample collection were all anonymized. All participants provided verbal consent prior to participation.

## AUTHOR CONTRIBUTIONS

Jieming Qu and Min Zhou first drew up the study plan and clarified the aim of the experiment. Jingwen Huang, Ying Xie, and Daoyi Yuan designed the experiments in detail, collected and analyzed data, and wrote the paper. Jingwen Huang and Lingxi Guo recruited participants and collected the clinical data and samples. Jingwen Huang, Ying Xie, and Min Zhou did the critical revision of the manuscript. Jieming Qu and Min Zhou are responsible for the integrity of the work as a whole. All authors approved the final version of the manuscript to be published.

## DATA AVAILABILITY STATEMENT

The data that support the findings of this study are available from the corresponding author upon reasonable request.

## Supporting information

Supporting Information

Supporting Information

Supporting Information

Supporting Information

Supporting Information

Supporting Information

Supporting Information

Supporting Information

Supporting Information

Supporting Information

Supporting Information

Supporting Information

Supporting Information
