# Supplementary material for: Identification of distinct metabolic characteristics of pneumonia in type 2 diabetes mellitus
Source: Clin Transl Med. 2021 Feb 4;11(2):e303. doi: 10.1002/ctm2.303 (PMC7862164; doi:10.1002/ctm2.303)
Supplement: Supplementary file 1 — Supporting Information [file CTM2-11-e303-s001.docx]

**Supplemental Table 1.** Chemometric parameters for the cross-validation of the OPLS-DA model.

| Analytical tool | A | R2X(cum) | R2Y(cum) | Q2(cum) |  |
| --- | --- | --- | --- | --- | --- |
| **Organic extract, ESI positive mode** | | | | | |
| Healthy controls and pneumonia patients with T2DM | 3 | 0.615 | 0.867 | 0.806 |  |
| Pneumonia patients with T2DM and T2DM patients without pneumonia | 3 | 0.628 | 0.88 | 0.834 |  |
| **Organic extract, ESI negative mode** | | | | | |
| Healthy controls and pneumonia patients with T2DM | 3 | 0.451 | 0.902 | 0.806 |  |
| Pneumonia patients with T2DM and T2DM patients without pneumonia | 3 | 0.490 | 0.903 | 0.805 |  |
| **Aqueous extract, ESI positive mode** | | | | | |
| Healthy controls and pneumonia patients with T2DM | 3 | 0.446 | 0.936 | 0.868 |  |
| Pneumonia patients with T2DM and T2DM patients without pneumonia | 3 | 0.398 | 0.913 | 0.808 |  |
| **Aqueous extract, ESI negative mode** | | | | | |
| Healthy controls and pneumonia patients with T2DM | 3 | 0.426 | 0.932 | 0.844 |  |
| Pneumonia patients with T2DM and T2DM patients without pneumonia | 3 | 0.437 | 0.908 | 0.797 |  |

The component number (A), R2X (cum), R2Y (cum) and Q2 (cum) were parameters of the cross-validation, which carried out a leave-one-out procedure to determine the quality and predictability of the model.
