# Supplementary material for: Identification of distinct metabolic characteristics of pneumonia in type 2 diabetes mellitus
Source: Clin Transl Med. 2021 Feb 4;11(2):e303. doi: 10.1002/ctm2.303 (PMC7862164; doi:10.1002/ctm2.303)
Supplement: Supplementary file 2 — Supporting Information [file CTM2-11-e303-s002.docx]

**Supplemental Table 2.** Identification of significant metabolites between pneumonia patients with T2DM and healthy subjects and pneumonia patients with T2DM and T2DM patients without pneumonia.

| **Metabolite** | **HMDB** | **Column** | **Mode** | **RT (min)** | **Extract mass** | **Change (S/H)*** | **Change (S/D)^#^** | **Pathway** |
| --- | --- | --- | --- | --- | --- | --- | --- | --- |
| PAF C-16 | HMDB0062689 | C18 | ESI+ | 8.84 | 281.662 | 0.596 | 0.555 | Glycerophospholipid metabolism |
| tetranor-12(S)-HETE | HMDB0060055 | C18 | ESI+ | 3.18 | 299.222 | 0.437 | 0.373 | Arachidonic acid metabolism |
| Methyl tetradecanoate | HMDB0030469 | C18 | ESI+ | 1.66 | 315.216 | 6.393 | 6.390 | Fatty acid metabolism |
| 1-Linoleoyl Glycerol | HMDB0011568 | C18 | ESI+ | 10.18 | 337.274 | 0.170 | 0.365 | Fatty acid metabolism |
| Leukotriene B4 | HMDB0001085 | C18 | ESI+ | 2.9 | 343.248 | 11.366 | 12.507 | Arachidonic acid metabolism |
| Prostaglandin F2a | HMDB0001139 | C18 | ESI+ | 2.85 | 361.257 | 11.356 | 9.850 | Arachidonic acid metabolism |
| PGG2 | HMDB0003235 | C18 | ESI+ | 2.08 | 369.224 | 5.035 | 5.639 | Arachidonic acid metabolism |
| HDoHE | HMDB0060049 | C18 | ESI+ | 10.16 | 377.267 | 0.524 | 0.622 | Fatty acid metabolism |
| Heptadecanoyl carnitine | HMDB0006210 | C18 | ESI+ | 2.9 | 414.169 | 10.392 | 11.318 | Fatty acid metabolism |
| Leukotriene E4 | HMDB0002200 | C18 | ESI+ | 8.01 | 457.280 | 3.628 | 3.281 | Arachidonic acid metabolism |
| LysoPE(0:0/18:0) | HMDB0011129 | C18 | ESI+ | 8.01 | 464.314 | 0.680 | 0.739 | Glycerophospholipid metabolism |
| LysoPC(P-18:0) | HMDB0013122 | C18 | ESI+ | 9.89 | 508.376 | 0.696 | 0.736 | Glycerophospholipid metabolism |
| LysoPC(17:0) | HMDB0012108 | C18 | ESI+ | 7.86 | 510.356 | 0.467 | 0.556 | Glycerophospholipid metabolism |
| PC(16:0/P-16:0) | HMDB0007994 | C18 | ESI+ | 6.52 | 518.322 | 0.658 | 0.678 | Glycerophospholipid metabolism |
| PAF C-16 | HMDB0062689 | C18 | ESI+ | 9.29 | 524.371 | 0.548 | 0.568 | Glycerophospholipid metabolism |
| PA(17:0/0:0) | HMDB0062318 | C18 | ESI+ | 8.14 | 532.338 | 0.439 | 0.508 | Glycerophospholipid metabolism |
| LysoPE(22:0/0:0) | HMDB0011520 | C18 | ESI+ | 9.96 | 538.386 | 0.629 | 0.681 | Glycerophospholipid metabolism |
| LPC(20:4) | HMDB0010395 | C18 | ESI+ | 7.51 | 544.339 | 0.750 | 0.725 | Glycerophospholipid metabolism |
| PAF C-16 | HMDB0011128 | C18 | ESI+ | 8.82 | 546.353 | 0.515 | 0.553 | Glycerophospholipid metabolism |
| LysoPC(20:2) | HMDB0010392 | C18 | ESI+ | 8.1 | 548.372 | 0.588 | 0.663 | Glycerophospholipid metabolism |
| LPC(22:5) | HMDB0010402 | C18 | ESI+ | 8.1 | 570.353 | 0.530 | 0.584 | Glycerophospholipid metabolism |
| PA(18:0/18:3) | HMDB0114878 | C18 | ESI+ | 25.81 | 740.523 | 1.550 | 1.702 | Glycerophospholipid metabolism |
| DG(42:6) | HMDB07409 | C18 | ESI+ | 25.82 | 760.583 | 1.484 | 1.479 | Glycerophospholipid metabolism |
| DG(44:10) | HMDB0007701 | C18 | ESI+ | 25.52 | 780.551 | 1.385 | 1.273 | Glycerophospholipid metabolism |
| Tyramine | HMDB0000306 | T3 | ESI+ | 3.23 | 120.081 | 0.852 | 0.806 | Tyrosine metabolism |
| Succinic acid semialdehyde | HMDB0001259 | T3 | ESI+ | 22.56 | 140.996 | 0.881 | 0.856 | Glutamate metabolism |
| trans-S-(1-Propenyl)-L-cysteine | HMDB0029440 | T3 | ESI+ | 2.58 | 162.058 | 1.524 | 2.017 | Cysteine metabolism |
| D-(+)-Malic acid | HMDB0031518 | T3 | ESI+ | 23.45 | 172.989 | 0.872 | 0.681 | Fatty acid metabolism |
| 20-carboxy-LTB4 | HMDB0006059 | T3 | ESI+ | 5.53 | 195.099 | 1.300 | 1.419 | Arachidonic acid metabolism |
| S-(Allylthio)-L-cysteine | HMDB0038669 | T3 | ESI+ | 2.94 | 211.057 | 1.264 | 1.251 | Cysteine metabolism |
| D-Biotin | HMDB0000030 | T3 | ESI+ | 4.77 | 227.088 | 1.223 | 1.228 | Biotin metabolism |
| Traumatic Acid | HMDB0000933 | T3 | ESI+ | 5.89 | 229.143 | 1.179 | 1.177 | Fatty acid metabolism |
| 12-Hydroxydodecanoic acid | HMDB0002059 | T3 | ESI+ | 1.08 | 239.160 | 0.855 | 0.797 | Fatty acid metabolism |
| CMPF | HMDB0061112 | T3 | ESI+ | 5.35 | 241.104 | 1.396 | 1.698 | Lipid peroxidation |
| Matricarin | HMDB0035790 | T3 | ESI+ | 5.99 | 269.135 | 1.148 | 1.139 | Lipid peroxidation |
| Histidinyl-Asparagine | HMDB0028880 | T3 | ESI+ | 5.59 | 311.146 | 1.196 | 1.161 | Dipeptide |
| Tsangane L 3-glucoside | HMDB0040824 | T3 | ESI+ | 7.71 | 339.214 | 1.368 | 1.451 | Fatty acid metabolism |
| 19(R)-hydroxy-PGB2 | HMDB0004236 | T3 | ESI+ | 6.91 | 357.227 | 1.311 | 1.328 | Arachidonic acid metabolism |
| 3-hydroxypentadecanoyl carnitine | HMDB0061641 | T3 | ESI+ | 6.43 | 384.308 | 0.800 | 0.732 | Fatty acid metabolism |
| Taraxacolide 1-O-b-D-glucopyranoside | HMDB0035610 | T3 | ESI+ | 6.71 | 411.198 | 1.666 | 1.901 | Fatty acid metabolism |
| Phenethyl rutinoside | HMDB0032622 | T3 | ESI+ | 6.07 | 413.177 | 1.325 | 1.323 | Lipid metabolism |
| Cynaroside A | HMDB0040532 | T3 | ESI+ | 6.33 | 427.194 | 1.392 | 1.490 | Lipid peroxidation |
| Sphingosine-1-phosphate | HMDB0000277 | T3 | ESI+ | 6.81 | 443.263 | 1.302 | 1.469 | Phospholipid metabolism |
| PGP(27:0) | HMDB0116520 | T3 | ESI+ | 8.14 | 697.378 | 1.290 | 1.340 | Phospholipid metabolism |
| (S)-3-Hydroxybutyric acid | HMDB0000442 | T3 | ESI- | 2.44 | 103.041 | 1.888 | 2.305 | Ketone metabolism |
| 2-Ethylidenehexanal | HMDB0037152 | T3 | ESI- | 5.66 | 171.102 | 0.552 | 0.552 |  |
| 4-Hydroxybenzenesulfonic acid | HMDB0060015 | T3 | ESI- | 3.71 | 172.991 | 0.190 | 0.148 | Sulfate/Sulfite Metabolism |
| N-Acetylornithine | HMDB0003357 | T3 | ESI- | 1.8 | 173.092 | 0.150 | 0.160 | Amino acid metabolism |
| Dimethyl adipate | HMDB0041606 | T3 | ESI- | 5.26 | 173.081 | 0.461 | 0.456 | Fatty acid metabolism |
| 2,5-Diethyltetrahydrofuran | HMDB0029574 | T3 | ESI- | 5.84 | 173.118 | 0.510 | 0.516 |  |
| 5-Hydroxyindoleacetic acid | HMDB0000763 | T3 | ESI- | 2.95 | 190.050 | 1.675 | 1.552 | Tryptophan metabolism |
| Leucyl-Alanine | HMDB0028922 | T3 | ESI- | 3.44 | 201.077 | 0.533 | 0.488 | Dipeptide |
| Ethyl 3-oxohexanoate | HMDB0031307 | T3 | ESI- | 4.76 | 203.093 | 0.475 | 0.436 | carboxylic acid derivative |
| Pyrogallol-1-O-sulphate Pyrogallol-2-O-sulphate | HMDB0060016 | T3 | ESI- | 2.85 | 204.980 | 0.044 | 0.111 |  |
| Kynurenine | HMDB0000684 | T3 | ESI- | 2.96 | 207.077 | 1.673 | 1.574 | Tryptophan Metabolism |
| Genipic acid | HMDB0036072 | T3 | ESI- | 2.43 | 229.068 | 2.155 | 3.600 | Cell signaling |
| (E)-2-Methyl-2-buten-1-ol O-beta-D-Glucopyranoside | HMDB0033064 | T3 | ESI- | 4.41 | 229.107 | 0.569 | 0.557 | Fatty acid metabolism |
| Asparaginyl-Valine | HMDB0028744 | T3 | ESI- | 2.1 | 230.114 | 0.161 | 0.156 | Dipeptide |
| Phaseolic acid | HMDB0031897 | T3 | ESI- | 4.96 | 261.134 | 0.613 | 0.619 | Fatty acid metabolism |
| Menthone 1,3-glyceryl ketal | HMDB0040004 | T3 | ESI- | 6.4 | 273.170 | 0.600 | 0.636 | Fatty acid metabolism |
| Dipropyl hexanedioate | HMDB0041614 | T3 | ESI- | 5.39 | 275.149 | 0.521 | 0.564 | Fatty acid metabolism |
| (3S,5R,6R,7E)-3,5,6-Trihydroxy-7-megastigmen-9-one | HMDB0038736 | T3 | ESI- | 5.58 | 287.149 | 0.526 | 0.560 | Fatty acid metabolism |
| 9-Oxohexadecanoic acid | HMDB0030973 | T3 | ESI- | 7.84 | 315.216 | 0.567 | 0.571 | Fatty acid metabolism |
| Ribose-1-arsenate | HMDB0012285 | T3 | ESI- | 2.37 | 318.963 | 1.578 | 1.920 | Nicotinamide metabolism |
| 16-Hydroxy-10-oxohexadecanoic acid | HMDB0041287 | T3 | ESI- | 6.43 | 331.212 | 0.588 | 0.566 | Fatty acid metabolism |
| 3,7,8,15-Scirpenetetrol | HMDB0037560 | T3 | ESI- | 5.39 | 343.136 | 0.673 | 0.716 | Fatty acid metabolism |
| MYCOPHENOLIC ACID | HMDB0015159 | T3 | ESI- | 2.37 | 355.096 | 2.545 | 3.338 | Mycophenolic Acid Metabolism |
| 20-hydroxy-PGF2α | HMDB0002886 | T3 | ESI- | 7.3 | 415.232 | 0.607 | 0.607 | Arachidonic acid metabolism |
| Suxibuzone | HMDB0042019 | T3 | ESI- | 5.72 | 419.165 | 0.250 | 0.254 | Fatty acid metabolism |
| Artemetin | HMDB0030095 | T3 | ESI- | 2.38 | 423.082 | 2.033 | 2.677 |  |
| Isohumulinone A | HMDB0030045 | T3 | ESI- | 6.98 | 423.198 | 0.413 | 0.426 | Fatty acid metabolism |
| 6-Ketoprostaglandin E1 | HMDB0004241 | T3 | ESI- | 6.47 | 431.228 | 0.493 | 0.451 | Arachidonic acid metabolism |
| PC(16:0/0:0)[U] / PC(16:0/0:0) | HMDB0010382 | T3 | ESI- | 11.16 | 540.329 | 0.470 | 0.461 | Glycerophospholipid metabolism |
| Hypoxanthine | HMDB0000157 | T3 | ESI+ | 1.43 | 137.045 | 0.633 | 0.650 | Purine Metabolism |
| 7a-Hydroxy-5b-cholestan-3-one | HMDB0006892 | T3 | ESI+ | 7.8 | 385.310 | 0.554 | 0.525 | Fatty acid metabolism |
| α-CEHC | HMDB0001518 | T3 | ESI- | 5.96 | 313.125 | 0.727 | 0.756 | Vitamin metabolism |
| Cysteinyl-Aspartate | HMDB0028771 | T3 | ESI- | 3.05 | 255.047 | 0.734 | 0.722 | Dipeptide |
| myo-Inositol | HMDB0000211 | T3 | ESI- | 0.8 | 215.032 | 1.380 | 1.617 | Inositol metabolic |
| 2-hydroxy-2-methyl-butyric acid | HMDB0001987 | T3 | ESI- | 3.18 | 117.056 | 1.687 | 1.743 | Fatty acid metabolism |
| Pyroglutamic acid | HMDB0000267 | T3 | ESI- | 1.19 | 128.035 | 1.390 | 1.315 | Glutathione metabolism |
| L-Phenylalanine | HMDB0000159 | T3 | ESI- | 3.2 | 164.071 | 1.263 | 1.234 | Phenylalanine metabolism |
| D-Fructose | HMDB0000660 | T3 | ESI- | 2.04 | 215.032 | 1.508 | 1.910 | Glucose homeostasis |
| Artemidinol | HMDB0030647 | T3 | ESI- | 1.98 | 215.072 | 2.289 | 2.077 |  |
| Inosine | HMDB0000195 | T3 | ESI- | 2.64 | 267.073 | 2.897 | 2.675 | Purine Metabolism |
| Xanthosine | HMDB0000299 | T3 | ESI- | 3.18 | 283.067 | 1.626 | 1.583 | Purine Metabolism |
| L-Homocystine | HMDB0000676 | T3 | ESI- | 1.18 | 313.051 | 1.455 | 1.515 | Amino acid metabolism |
| 1-Heptadecanoyl-glycerophosphoethanolamine | HMDB0061691 | C18 | ESI- | 5.34 | 526.315 | 0.527 | 0.536 | Glycerophospholipid metabolism |
| LPC(18:0) | HMDB0011149 | C18 | ESI- | 8.16 | 554.345 | 0.504 | 0.548 | Glycerophospholipid metabolism |
| Panaxydol linoleate | HMDB0041205 | C18 | ESI- | 8.24 | 557.370 | 0.690 | 0.776 | Fatty acid metabolism |
| LysoPE(20:0/0:0) | HMDB0011511 | C18 | ESI- | 8.79 | 568.361 | 0.573 | 0.570 | Glycerophospholipid metabolism |
| Asparagoside B | HMDB0029315 | C18 | ESI- | 4.78 | 577.374 | 3.716 | 3.810 | Fatty acid metabolism |
| Janthitrem C | HMDB0040684 | C18 | ESI- | 5.27 | 606.303 | 0.634 | 0.702 | Cell signaling |

* Relative metabolite concentrations in pneumonia patients with T2DM (S) compared to healthy subjects (H).

^#^ Relative metabolite concentrations in pneumonia patients with T2DM (S) compared to T2DM patients without pneumonia (D).
