# Supplementary material for: Identification of distinct metabolic characteristics of pneumonia in type 2 diabetes mellitus
Source: Clin Transl Med. 2021 Feb 4;11(2):e303. doi: 10.1002/ctm2.303 (PMC7862164; doi:10.1002/ctm2.303)
Supplement: Supplementary file 3 — Supporting Information [file CTM2-11-e303-s003.docx]

**Supplemental Table 3.** The detailed results from the MetaboAnalyst pathway analysis of different metabolites from pneumonia patients with T2DM vs. healthy subjects or T2DM patients without pneumonia.

|  | Total | Expected | Hits | Raw p | #NAME? | Holm adjust | FDR | Impact |
| --- | --- | --- | --- | --- | --- | --- | --- | --- |
| **Phenylalanine, tyrosine and tryptophan biosynthesis** | 4 | 0.087742 | 1 | 0.084976 | 2.4654 | 1 | 1 | 0.5 |
| **Phenylalanine metabolism** | 10 | 0.21935 | 1 | 0.19945 | 1.6122 | 1 | 1 | 0.35714 |
| **Biotin metabolism** | 10 | 0.21935 | 1 | 0.19945 | 1.6122 | 1 | 1 | 0.2 |
| **Ether lipid metabolism** | 20 | 0.43871 | 1 | 0.36005 | 1.0215 | 1 | 1 | 0.14458 |
| **Inositol phosphate metabolism** | 30 | 0.65806 | 1 | 0.48919 | 0.715 | 1 | 1 | 0.12939 |
| **Arachidonic acid metabolism** | 36 | 0.78968 | 2 | 0.18563 | 1.684 | 1 | 1 | 0.1166 |
| **Glycerophospholipid metabolism** | 36 | 0.78968 | 2 | 0.18563 | 1.684 | 1 | 1 | 0.11182 |
| **Tryptophan metabolism** | 41 | 0.89935 | 2 | 0.22622 | 1.4862 | 1 | 1 | 0.10807 |
| Alanine, aspartate and glutamate metabolism | 28 | 0.61419 | 1 | 0.46558 | 0.76448 | 1 | 1 | 0.04808 |
| Primary bile acid biosynthesis | 46 | 1.009 | 1 | 0.64497 | 0.43856 | 1 | 1 | 0.04135 |
| Phosphatidylinositol signaling system | 28 | 0.61419 | 1 | 0.46558 | 0.76448 | 1 | 1 | 0.03736 |
| Butanoate metabolism | 15 | 0.32903 | 1 | 0.28411 | 1.2584 | 1 | 1 | 0.03175 |
| Tyrosine metabolism | 42 | 0.92129 | 1 | 0.61102 | 0.49262 | 1 | 1 | 0.02463 |
| Sphingolipid metabolism | 21 | 0.46065 | 1 | 0.37427 | 0.98277 | 1 | 1 | 0.02434 |
| Purine metabolism | 65 | 1.4258 | 3 | 0.16798 | 1.7839 | 1 | 1 | 0.01885 |

The Total is the total number of compounds in the pathway; the Hits is the actually matched number from the user uploaded data; the Impact is the pathway impact value calculated from pathway topology analysis. Most reliably affected biological pathways are bold in the tables. These pathways have an impact > 0.1.
