# Supplementary material for: Identification of distinct metabolic characteristics of pneumonia in type 2 diabetes mellitus
Source: Clin Transl Med. 2021 Feb 4;11(2):e303. doi: 10.1002/ctm2.303 (PMC7862164; doi:10.1002/ctm2.303)
Supplement: Supplementary file 4 — Supporting Information [file CTM2-11-e303-s004.docx]

**Supplemental Table 4**. Identification of differential metabolites between pneumonia patients with T2DM and T2DM patients without pneumonia or healthy controls in the discovery set.

| Metabolite | HMDB | Fold Change  (S/H)^*^ | AUC | Fold Change  (S/D)^#^ | AUC |
| --- | --- | --- | --- | --- | --- |
| Asparaginyl-Valine | HMDB0028744 | 0.161 | 0.941 | 0.156 | 0.937 |
| Dimethyl adipate | HMDB0041606 | 0.461 | 0.981 | 0.456 | 0.978 |
| PA(17:0/0:0) | HMDB0062318 | 0.439 | 0.973 | 0.508 | 0.904 |
| LPC(18:0） | HMDB0011149 | 0.504 | 0.952 | 0.548 | 0.923 |
| 2,5-Diethyltetrahydrofuran | HMDB0029574 | 0.510 | 0.911 | 0.516 | 0.936 |
| PAF C-16 | HMDB0011128 | 0.515 | 0.887 | 0.553 | 0.880 |
| (3S,5R,6R,7E)-3,5,6-Trihydroxy-7-megastigmen-9-one | HMDB0038736 | 0.526 | 0.897 | 0.560 | 0.925 |
| 1-Heptadecanoylglycerophosphoethanolamine | HMDB0061691 | 0.527 | 0.871 | 0.536 | 0.852 |
| LPC(22:5) | HMDB0010402 | 0.530 | 0.888 | 0.584 | 0.864 |
| 9-Oxohexadecanoic acid | HMDB0030973 | 0.567 | 0.950 | 0.571 | 0.959 |
| LPE(20:0/0:0) | HMDB0011511 | 0.573 | 0.907 | 0.570 | 0.925 |
| PC(16:0/P-16:0) | HMDB0007994 | 0.658 | 0.928 | 0.678 | 0.955 |
| Ribose-1-arsenate | HMDB0012285 | 1.578 | 0.880 | 1.920 | 0.955 |
| (S)-3-Hydroxybutyric acid | HMDB0000442 | 1.888 | 0.893 | 2.305 | 0.961 |
| Genipic acid | HMDB0036072 | 2.155 | 0.861 | 3.600 | 0.948 |
| Artemidinol | HMDB0030647 | 2.289 | 0.864 | 2.077 | 0.872 |
| Leukotriene E4 | HMDB0002200 | 3.628 | 0.889 | 3.281 | 0.882 |
| Asparagoside B | HMDB0029315 | 3.716 | 0.975 | 3.810 | 0.984 |
| Prostaglandin G2 | HMDB0003235 | 5.035 | 0.918 | 5.639 | 0.944 |
| Methyl tetradecanoate | HMDB0030469 | 6.393 | 0.986 | 6.390 | 0.993 |
| Heptadecanoyl carnitine | HMDB0006210 | 10.392 | 0.970 | 11.318 | 0.975 |
| Prostaglandin F2α | HMDB0001139 | 11.356 | 0.987 | 9.850 | 0.978 |
| Leukotriene B4 | HMDB0001085 | 11.366 | 0.980 | 12.507 | 0.980 |

* Relative metabolite concentrations in pneumonia patients with T2DM (S) compared to healthy subjects (H).

^#^ Relative metabolite concentrations in pneumonia patients with T2DM (S) compared to T2DM patients without pneumonia (D).
