# Supplementary material for: Identification of distinct metabolic characteristics of pneumonia in type 2 diabetes mellitus
Source: Clin Transl Med. 2021 Feb 4;11(2):e303. doi: 10.1002/ctm2.303 (PMC7862164; doi:10.1002/ctm2.303)
Supplement: Supplementary file 5 — Supporting Information [file CTM2-11-e303-s005.docx]

**Supplemental Table 5.** Identification of differential metabolites between pneumonia patients with T2DM and T2DM patients without pneumonia or healthy controls in the validation set.

| Metabolite | HMDB | Fold Change  (S/H)^*^ | AUC | Fold Change  (S/D)^#^ | AUC |
| --- | --- | --- | --- | --- | --- |
| Asparaginyl-Valine | HMDB0028744 | 1.950 | 0.846 | 1.377 | 0.688 |
| Dimethyl adipate | HMDB0041606 | 0.660 | 0.661 | 0.569 | 0.688 |
| PA(17:0/0:0) | HMDB0062318 | 0.655 | 0.816 | 0.699 | 0.774 |
| LPC(18:0） | HMDB0011149 | 0.604 | 0.836 | 0.662 | 0.794 |
| 2,5-Diethyltetrahydrofuran | HMDB0029574 | 0.568 | 0.776 | 0.472 | 0.831 |
| PAF C-16 | HMDB0011128 | 0.416 | 0.729 | 1.452 | 0.738 |
| (3S,5R,6R,7E)-3,5,6-Trihydroxy-7-megastigmen-9-one | HMDB0038736 | 0.564 | 0.905 | 0.459 | 0.958 |
| 1-Heptadecanoylglycerophosphoethanolamine | HMDB0061691 | 0.562 | 0.856 | 0.645 | 0.823 |
| LPC(22:5) | HMDB0010402 | 0.689 | 0.813 | 0.757 | 0.740 |
| 9-Oxohexadecanoic acid | HMDB0030973 | 0.442 | 0.889 | 0.337 | 0.955 |
| LPE(20:0/0:0) | HMDB0011511 | 0.636 | 0.910 | 0.672 | 0.874 |
| PC(16:0/P-16:0) | HMDB0007994 | 1.209 | 0.753 | 1.147 | 0.675 |
| Ribose-1-arsenate | HMDB0012285 | 1.178 | 0.677 | 1.197 | 0.694 |
| (S)-3-Hydroxybutyric acid | HMDB0000442 | 2.017 | 0.858 | 1.432 | 0.754 |
| Genipic acid | HMDB0036072 | 2.554 | 0.874 | 1.547 | 0.729 |
| Artemidinol | HMDB0030647 | 2.006 | 0.913 | 1.495 | 0.811 |
| Leukotriene E4 | HMDB0002200 | 1.431 | 0.575 | 1.722 | 0.643 |
| Asparagoside B | HMDB0029315 | 1.226 | 0.674 | 1.133 | 0.554 |
| Prostaglandin G2 | HMDB0003235 | 1.068 | 0.532 | 1.112 | 0.518 |
| Methyl tetradecanoate | HMDB0030469 | 1.902 | 0.699 | 1.894 | 0.692 |
| Heptadecanoyl carnitine | HMDB0006210 | 3.046 | 0.804 | 3.065 | 0.815 |
| Prostaglandin F2α | HMDB0001139 | 4.031 | 0.661 | 3.867 | 0.662 |
| Leukotriene B4 | HMDB0001085 | 3.629 | 0.650 | 4.207 | 0.671 |

* Relative metabolite concentrations in pneumonia patients with T2DM (S) compared to healthy subjects (H).

^#^ Relative metabolite concentrations in pneumonia patients with T2DM (S) compared to T2DM patients without pneumonia (D).
