# Supplementary material for: Identification of distinct metabolic characteristics of pneumonia in type 2 diabetes mellitus
Source: Clin Transl Med. 2021 Feb 4;11(2):e303. doi: 10.1002/ctm2.303 (PMC7862164; doi:10.1002/ctm2.303)
Supplement: Supplementary file 6 — Supporting Information [file CTM2-11-e303-s006.docx]

**Supplemental Table 6**. Clinical information of the subjects enrolled in transcriptomics analysis.

| Baseline characteristic | Healthy controls  (n=6) | T2DM patients  (n=6) | Pneumonia patients with T2DM  (n=6) | P Value |
| --- | --- | --- | --- | --- |
| Age, years | 65.50[57.25, 75.75] | 67.00[57.70,80.00] | 61.00[52.50,66.50] | 0.920 |
| Male (n,%) | 4(66.7) | 4(66.7) | 5(83.3) | 0.758 |
| BMI, kg·m^-2^ | 23.96[22.33,24.96] | 23.35[20.92,24.51] | 23.60[22.23,24.64] | 0.950 |
| Leucocyte (×10^9^/L) | 7.25[3.90,8.48] | 6.35[6.05,6.83] | 7.48[5.3910.02] | 0.403 |
| Neutrophil (×10^9^/L) | 4.75[2.18,5.50] | 3.56[3.12,3.96] | 4.97[3.25,7.93] | 0.199 |
| Lymphocyte(×10^9^/L) | 1.50[0.56,2.09] | 2.17[1.70,2.74] | 1.28[0.70,2.32] | 0.119 |
| Neutrophil, % | 62.35[55.03,65.45] | 55.90[47.78,63.95] | 69.05[54.35,79.20] | 0.151 |
| Lymphocyte,% | 24.40[15.35,30.08] | 33.20[27.95,41.83] | 16.70[7.13,38.85] | 0.151 |
| Albumin(g/L) | 42.50[41.00,44.50] | 42.50[39.50,44.75] | 40.00[31.50,42.00] | **0.021** |
| Creatinine(μmol/L) | 91.00[72.50,107.75] | 85.50[65.00,104.75] | 74.00[60.00,79.50] | 0.610 |
| Urea nitrogen(mmol/L) | 4.75[3.05,6.20] | 6.15[4.23,7.45] | 6.95[5.40,10.08] | 0.114 |
| Glucose (mmol/L) | 5.49[4.92,5.89] | 8.16[7.57,9.43] | 7.67[4.81,15.02] | **0.046** |

Data are presented as median (interquartile range) for continuous variables and n (%) for categorical variables.
