# Supplementary material for: Identification of distinct metabolic characteristics of pneumonia in type 2 diabetes mellitus
Source: Clin Transl Med. 2021 Feb 4;11(2):e303. doi: 10.1002/ctm2.303 (PMC7862164; doi:10.1002/ctm2.303)
Supplement: Supplementary file 7 — Supporting Information [file CTM2-11-e303-s007.docx]

**Supplemental Table 7**. The detailed results from the MetaboAnalyst joint pathway analysis of different metabolites and genes from pneumonia patients with T2DM vs. healthy subjects.

|  | Total | Expected | Hits | Raw p | #NAME? | Holm adjust | FDR | Impact |
| --- | --- | --- | --- | --- | --- | --- | --- | --- |
| Glycerophospholipid metabolism | 86 | 7.4016 | 19 | 7.71E-05 | 9.471 | 0.006472 | 0.006472 | 0.75294 |
| Neomycin, kanamycin and gentamicin biosynthesis | 4 | 0.34426 | 3 | 0.002357 | 6.0506 | 0.19559 | 0.066098 | 0.66667 |
| Amino sugar and nucleotide sugar metabolism | 79 | 6.7992 | 15 | 0.002361 | 6.0488 | 0.19559 | 0.066098 | 0.58974 |
| Glycerolipid metabolism | 35 | 3.0123 | 8 | 0.008066 | 4.8202 | 0.65331 | 0.12917 | 0.55882 |
| Mucin type O-glycan biosynthesis | 22 | 1.8934 | 6 | 0.008724 | 4.7417 | 0.69792 | 0.12917 | 0.52381 |
| Starch and sucrose metabolism | 43 | 3.7008 | 9 | 0.009227 | 4.6857 | 0.7289 | 0.12917 | 0.54762 |
| Phosphatidylinositol signaling system | 74 | 6.3689 | 12 | 0.021932 | 3.8198 | 1 | 0.26319 | 0.64384 |
| Galactose metabolism | 51 | 4.3893 | 9 | 0.02726 | 3.6023 | 1 | 0.28623 | 0.34 |
| Nitrogen metabolism | 10 | 0.86066 | 3 | 0.047924 | 3.0381 | 1 | 0.42855 | 0.22222 |
| Fructose and mannose metabolism | 40 | 3.4426 | 7 | 0.051017 | 2.9756 | 1 | 0.42855 | 0.35897 |
| Pantothenate and CoA biosynthesis | 34 | 2.9262 | 6 | 0.066305 | 2.7135 | 1 | 0.50633 | 0.42424 |
| beta-Alanine metabolism | 44 | 3.7869 | 7 | 0.078502 | 2.5446 | 1 | 0.54951 | 0.4186 |
| Ascorbate and aldarate metabolism | 13 | 1.1189 | 3 | 0.094437 | 2.3598 | 1 | 0.57752 | 0.41667 |

The Total is the total number of compounds and genes in the pathway; the Hits is the actually matched number from the user uploaded data; the Impact is the pathway impact value calculated from pathway topology analysis. 13 most reliably affected biological pathways in both metabolomics and transcriptomics are presented.
