# Supplementary material for: Identification of distinct metabolic characteristics of pneumonia in type 2 diabetes mellitus
Source: Clin Transl Med. 2021 Feb 4;11(2):e303. doi: 10.1002/ctm2.303 (PMC7862164; doi:10.1002/ctm2.303)
Supplement: Supplementary file 8 — Supporting Information [file CTM2-11-e303-s008.docx]

**Table S8**. Logistic regression model based on serum LPC (18:0) and glucose level between the pneumonia patients with or without T2DM.

|  | P Value | β estimates |
| --- | --- | --- |
| LPC (18:0) | 0.013 | -0.846 |
| Glucose level | <0.0001 | 0.777 |
