# Supplementary figures and images for: Identification of distinct metabolic characteristics of pneumonia in type 2 diabetes mellitus
Source: Clin Transl Med. 2021 Feb 4;11(2):e303. doi: 10.1002/ctm2.303 (PMC7862164; doi:10.1002/ctm2.303)

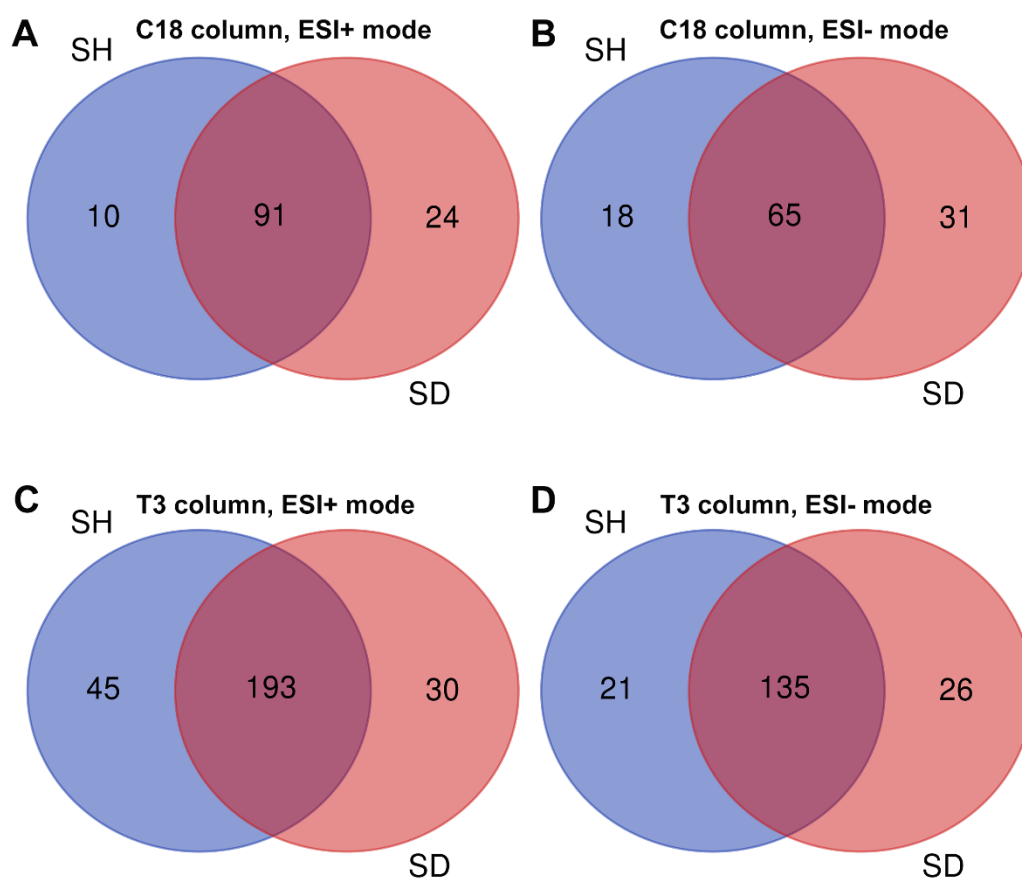

**Supplementary Figure 1**

Supplement: Supplementary file 9 — Supporting Information [file CTM2-11-e303-s009.pdf]

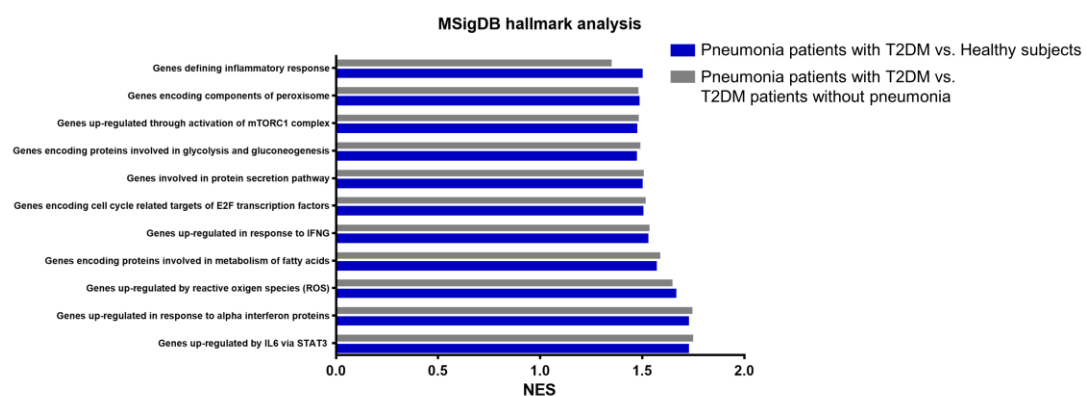

Supplementary Figure 2

Supplement: Supplementary file 10 — Supporting Information [file CTM2-11-e303-s010.pdf]
