# Supplementary material for: Identification of distinct metabolic characteristics of pneumonia in type 2 diabetes mellitus
Source: Clin Transl Med. 2021 Feb 4;11(2):e303. doi: 10.1002/ctm2.303 (PMC7862164; doi:10.1002/ctm2.303)
Supplement: Supplementary file 12 — Supporting Information [file CTM2-11-e303-s011.docx]

**Supplementary Figure 1**

Venn diagram of the peak features that were differentially identified between pneumonia patients with T2DM and T2DM patients without pneumonia or healthy controls.

S represents the peak features that identified in pneumonia patients with T2DM; D represents the peak features that identified in T2DM patients without pneumonia; H represents the peak features that identified in healthy subjects.

**Supplementary Figure 2**

GSEA analysis for the differentially regulated pathway between pneumonia patients with T2DM group and T2DM patients without pneumonia group or healthy subject group.

NES, Normalized Enrichment Score.

**Supplementary Figure 3**

Analysis on the differential genes

(A) Venn diagram of the differential genes that were identified between pneumonia patients with T2DM and T2DM patients without pneumonia or healthy controls (B) Transcriptional responses on the glycerophospholipid metabolism of T2DM patients with pneumonia in relation to healthy subjects. Differentially regulated genes are shown in colored frames: red for upregulation, green for downregulation.
