# Supplementary material for: Identification of distinct metabolic characteristics of pneumonia in type 2 diabetes mellitus
Source: Clin Transl Med. 2021 Feb 4;11(2):e303. doi: 10.1002/ctm2.303 (PMC7862164; doi:10.1002/ctm2.303)
Supplement: Supplementary file 13 — Supporting Information [file CTM2-11-e303-s012.docx]

**Supplementary Materials and Methods**

**Patient data**

The participants were all recruited from Shanghai Jiao Tong University of Medicine Affiliated Ruijin Hospital (Shanghai, China) between March 2018 and December 2019. During the same period, we recruited healthy control subjects from the physical examination center of Ruijin Hospital. All study subjects voluntarily enrolled in the study and provided written, informed consent prior to participation. A chest computed tomography scan was used to diagnose pneumonia. Patients with a history of cancer or with an autoimmune disease, or those receiving intravenous steroids or immunosuppressant treatment or were excluded. Clinical baseline characteristics were collected at admission or obtained within 24 h of admission and whole treatment courses were recorded. Samples of healthy subjects without pneumonia or T2DM and T2DM patients without pneumonia were obtained under no sign of infection, while samples of pneumonia patients with T2DM and non-diabetic pneumonia patients were obtained at first admission. The study protocol was approved by Ruijin Hospital Ethics Committee.

**Serum collection and preparation**

Antecubital venous blood samples (5 ml) were taken from consenting participants in the morning. After collection, the blood samples were immediately placed on ice for transportation and were processed within 3h (800 RCF, 4℃, 10 min), which were subsequently stored at −80°C[^1^](#_ENREF_1). 100 μL full thawed serum was mixed with 20 μL internal standard (100nM Cholic-2,2,4,4-d4 acid, 50nM LPC19:0 and 200μM L-phenylalanine-d2 were mixed equally ) and then 750 μL methanol and 2.5mL methyl tert-butyl ether (MTBE) were added successively. The mixture was thoroughly vortexed for 3 min both before and after MTBE addition. After incubating for 10 min on ice, 625 μL of Milli-Q water was added and vortexed for 3 min. The mixture was centrifuged at 4°C (13000 rpm for 10 minutes) to form a two-phase system. The organic extraction supernatant was transferred, dried in a Savant vacuum concentrator and resuspended in 100 μL acetonitrile/water (4:1, v/v) before analysis. The aqueous extraction precipitate was transferred and centrifuged at 13000rpm for 10 minutes at 4°C to take the supernatant, then dried and resuspended in 100 μL methanol/water (1:1, v/v) before analysis.

To verify the system conditions and data quality in the metabolic profiling process, pooled quality control samples (QC) were prepared by mixing five of the serum samples in the four groups (100 μl of each sample). Pretreatment of the quality control samples was in parallel with and identical to that of the study samples [^2^](#_ENREF_2).

**Metabolomic profiling based on UPLC-QTOF/MS**

Serum was immediately separated from the collected blood samples and analyzed using untargeted serum metabolomic profiling conducted on a 30AD UPLC system (Shimadzu, Kyoto, Japan) coupled to a TripleTOF 5600+ mass spectrometer system (AB SCIEX, Framingham, MA). An HSS T3 column (100 mm × 2.1 mm, 1.8 μm, Waters) was used to analyze the aqueous extract in the positive and negative electrospray ionization (ESI) mode. A BEH C18 column (100 mm × 2.1 mm, 1.7 μm, Waters) was used to analyze the organic extract in the positive and negative ESI mode. The mobile phases are water contain of 0.1% formic acid (A) and methanol (B) for aqueous extract analysis. The gradient program was: 0–1min 5% B, 1–5min 5%–75% B, 5–15min 75%–100% B, 15–21min 100% B, 21–23min 100%–5% B, 23–26min 5% B for equilibration. The mobile phases are water contain of 0.1% formic acid (A) and acetonitrile contain of 0.1% formic acid (B) for organic extract analysis. The gradient program was: 0–2min 50% B, 2–12min 50%–90% B, 12–15min 90%–100% B, 15–24min 100% B, 24–25min 100%–50% B, 25–28min 50% B for equilibration. The flow rate was 0.35 mL/min, and the oven temperature was kept at 40 °C. For each sample 5 μL was analyzed. Data were acquired from 50 to 1000 mass-to-charge ratio (m/z). The parameters were as follows: curtain gas(CUR), 35 psi; ion source gas 1 (GS1), 45 psi; ion source gas 2 (GS2), 45 psi; temperature(TEM), 550 °C; and ionspray voltage floating(ISVF), 5000 v.

**Data processing**

Before statistical analysis, pre-processing, peak ﬁltering, retention time correction, feature alignment, and normalization by total peak area were carried out. The parameters were as follows: signal to noise >5; retention time deviation <15 S; mass to charge ratio deviation <5 PPM. Extracted variables should have response chromatographic peaks in at least 80% of the samples for peak alignment and extraction of molecular characteristics. Before visualization of any metabolic clustering in different groups of samples, variables with a coefficient of variation > 30% in pooled quality control (QC) samples were excluded. The processed data were log-transformed and imported into SIMCA-P 13.0 (Umetrics AB, Umea, Sweden) for multivariate analysis.

Unsupervised principal component analysis (PCA) was used to filter outlying data and evaluate their reliability. A supervised model of orthogonal partial least-squares discriminant analysis (OPLS-DA) was then used to identify candidate biomarkers according to variable importance in projection (VIP) values and the S plot. Permutation testing with 200 replications was performed to assess the risk of model overfitting. A P value <0.05, VIP> 1 and the absolute value of P(corr) > 0.52 were used as the threshold for candidate metabolic biomarker selection. The identify of candidate biomarkers was using the Human Metabolome Database (HMDB; https://www.hmdb.ca/), METLIN (metlin.scripps.edu) and Lipid Maps (http://www.lipidmaps.org/). Results were compared with standard compounds in our laboratory or MS/MS profiles found in the above public database. Pathway analysis was performed by MetaboAnalyst (https://www.metaboanalyst.ca/) in order to clarify the functional significance of the observed changes in metabolite profiles.

**RNA isolation and transcriptomics analysis**

Peripheral blood mononuclear cells (PBMCs) were separated from peripheral blood using Lymphoprep™(density gradient: 1.077 ± 0.001 g/ml, Axis-Shield, Oslo, Norway) and then lysed for RNA isolation. Bulk RNA sequencing was performed by BGI America (Cambridge, MA, USA) using the BGISEQ-500 platform at a single-end read length of 50 bp and 30 × 10^6^ reads per sample. Gene Set Enrichment Analysis (GSEA) was performed to identify genes that showed consistent differences between diabetes with vs without pneumonia and healthy controls [^3^](#_ENREF_3). Gene Ontology and Molecular Signatures Database analysis were performed for transcription factor enrichment of differentially expressed genes based on false discovery rate < 0.05 [^4^](#_ENREF_4).

**Statistical analysis**

Continuous variables were expressed as median (interquartile range), and categorical variables were expressed as number and proportion as appropriate. Comparisons among 2 groups were performed by the student’s t-test or Mann-Whitney U test. Comparisons among more than 2 groups were performed by a one-way Kruskal-Wallis test or one-way analysis of variance. The Spearman or Pearson coefficient was calculated to assess correlations between variables depending on their non-normal or normal distribution. The p-values less than 0.05 were considered significant and values less than 0.01 were considered highly significant. Statistical analyses were performed using SPSS v26.0 (SPSS Inc, Chicago, IL, USA) and R studio v3.6 (https://www.r-project.org/) software programs.

**References**

1. Bernini P, Bertini I, Luchinat C, Nincheri P, Staderini S, Turano P. Standard operating procedures for pre-analytical handling of blood and urine for metabolomic studies and biobanks. *Journal of biomolecular NMR.* Apr 2011;49(3-4):231-243.

2. Dunn WB, Wilson ID, Nicholls AW, Broadhurst D. The importance of experimental design and QC samples in large-scale and MS-driven untargeted metabolomic studies of humans. *Bioanalysis.* Sep 2012;4(18):2249-2264.

3. Subramanian A, Tamayo P, Mootha VK, et al. Gene set enrichment analysis: a knowledge-based approach for interpreting genome-wide expression profiles. *Proceedings of the National Academy of Sciences of the United States of America.* Oct 25 2005;102(43):15545-15550.

4. Love MI, Huber W, Anders S. Moderated estimation of fold change and dispersion for RNA-seq data with DESeq2. *Genome biology.* 2014;15(12):550.
